# The healthcare experiences of women with cardiac disease in pregnancy and postpartum: A qualitative study

**DOI:** 10.1111/hex.13532

**Published:** 2022-05-26

**Authors:** Jane Hutchens, Jane Frawley, Elizabeth A. Sullivan

**Affiliations:** ^1^ School of Public Health, Faculty of Health University of Technology Sydney Ultimo New South Wales Australia; ^2^ College of Health, Mediicne and Wellbeing University of Newcastle Newcastle Australia; ^3^ Hunter Medical Research Institute Justice Health and Forensic Mental Health Network Newcastle Australia

**Keywords:** cardiac, patient experience, PCC, postpartum, pregnancy, qualitative

## Abstract

**Introduction:**

Cardiac disease affects an estimated 1%–4% of all pregnancies and is a leading cause of maternal morbidity and mortality. There is a lack of data on the healthcare experiences of affected women to inform health service delivery and person‐centred care. This study sought to explore and understand the healthcare experiences of women with cardiac disease in pregnancy and postpartum.

**Methods:**

This qualitative study used semi‐structured interviews with women who had cardiac disease in pregnancy or the first 12 months postpartum. Data were analysed using thematic analysis.

**Results:**

Participants were 25 women with pre‐existing or newly diagnosed acquired, genetic and congenital cardiac disease. Analysis of the interviews highlighted the discrepancy between care aspirations and experiences. The participants had a wide range of cardiac diseases and timing of diagnoses, but had similar healthcare experiences of being dismissed, not receiving the information they required, lack of continuity of care and clinical guidelines and of feeling out of place within a healthcare system that did not accommodate their combined needs as a mother and a cardiac patient.

**Conclusion:**

This study identified a lack of person‐centred care and responsiveness of the healthcare system in providing fit‐for‐purpose healthcare for women with complex disease who are pregnant or new mothers. In particular, cardiac and maternity care providers have an opportunity to listen to women who are the experts on their emergent healthcare needs, contributing to development of the knowledge base on the healthcare experiences of having cardiac disease in pregnancy and postpartum.

**Patient or Public Contribution:**

Public and patient input into the value and design of the study was gained through NSW Heart Foundation forums, including the Heart Foundation's women's patient group.

## 1 INTRODUCTION

Cardiac disease in pregnancy and postpartum (CDPP), pre‐existing or newly diagnosed cardiac disease in pregnancy or in the first 12 months postpartum, is associated with significant serious maternal morbidity and mortality.^1^, ^2^ CDPP includes a variety of structural heart and aortic diseases, cardiomyopathies, rhythm disorders, ischaemic heart disease and arterial dissections.

CDPP is under‐researched in Australia and internationally. Prevalence estimates range from 1% to 4%, with evidence of increasing prevalence due to delayed childbearing in middle‐ and high‐income countries, growing rates of cardio‐metabolic risk factors^3^ and increasing numbers of congenital heart disease survivors having children.^4^ Cardiovascular disease has been a leading medical cause of maternal death in Australia for the past five decades, responsible for 14.36% of all maternal deaths between 2009 and 2018.^5^ There is significant burden from maternal morbidity, with about one in four women with cardiac disease during pregnancy requiring hospitalization.^6^ As maternal mortality reduces, morbidity is increasing, and yet, current maternal morbidity monitoring is affected by inconsistent definitions and criteria, language and monitoring practices.^7^ The morbidity experienced by women encompasses physical, psychosocial, emotional and functional domains. It is necessary to establish evidence‐based information on cardiac presentations, cardiac‐related complications and women's experiences.

There is a lack of comprehensive data on the impact that a diagnosis of CDPP has on quality of life, psychosocial and emotional well‐being and the healthcare experiences of women during pregnancy and postpartum. A recent meta‐synthesis confirmed the paucity of research on the healthcare experiences of women with CDPP and highlighted the need for greater engagement with women and the development of models of care that are responsive to women's needs, knowledge and desired outcomes.^8^

Person‐centred care (PCC) is promoted as a model for improved patient outcomes and clinician satisfaction and is based upon the healthcare experiences and needs of patients.^9^ PCC protects a person's dignity, is respectful of, and responsive to, the preferences, needs and values of the individual, and is founded on mutual trust and understanding between the care‐giver and the recipient.^9^, ^10^

This study explores the healthcare experiences of women who had CDPP or the first 12 months postpartum in Australia, contributing to the knowledge base for developing guidelines and continuity of care frameworks, resources and PCC, thereby improving women's healthcare experiences and outcomes.

## 2 METHODS

### 2.1 Study design

A qualitative study was designed to examine women's experiences of CDPP, privilege women's voices, increase knowledge and improve clinical care and quality of life. Qualitative research focuses on the way people make sense of, and the meanings they ascribe to, their experiences and the world in which they live.^11^ A phenomenological perspective is adopted as is fitting for areas with little existing knowledge, and focuses on the commonality of subjective, lived experiences of a phenomenon within a particular group.^12^ The concept of the study was discussed with clinical and community groups from the NSW Heart Foundation. Ethics approval was granted by the University of Technology Sydney's *Human Research Ethics Committee (ETH19‐3372)*.

### 2.2 Participants and procedure

Criterion‐based purposive sampling was used to engage women who had a diagnosis of CDPP and were willing to participate in an in‐depth interview.^13^ Eligibility criteria specified mothers who have been diagnosed with cardiac disease before, during pregnancy or up to 1 year postpartum, living in Australia and who give birth to one or multiple babies beyond 20 weeks, gestation or 400 gm or greater birthweight. Women had to have adequate English fluency to participate in the interview.

The population we sought to interview was both ill‐defined and hard to reach due to a lack of prevalence data, and involved rare and uncommon conditions, limited registries and disease‐based support groups and, to our knowledge, no support groups specifically for CDPP.^14^ Online recruitment has been shown to be effective for recruitment for hard‐to‐reach groups^15^; therefore, we posted recruitment notices on the Facebook pages and groups of consenting cardiac groups and organizations, as well as via invitations distributed by cardiac support groups to members' emails and or group newsletters. Thirty‐three women responded, of whom 25 women fulfilled the inclusion criteria and agreed to proceed with an interview. Recruitment continued until we had adequate depth and breadth of data to sufficiently describe and analyse the participants' experiences and answer our research question.^16^

Most women lived in metropolitan areas, and of the four who lived in regional or rural areas, two transferred to metropolitan hospitals for care during their pregnancy or postpartum event. Fifteen women (60%) had tertiary‐level education, seven women (28%) had trade‐level education and three women (12%) had high school education. Their median age at interview was 39 years (range: 28–59). Participant characteristics are outlined in Table 1.

**Table 1:** Participant characteristics.

Category	Diagnosis	Timing of diagnosis	Age at diagnosis	Parity for first affected pregnancy
CHD	Tetralogy of Fallot	Pre‐existing	6 Weeks	1
CHD	Bicuspid aortic valve	Pre‐existing	26 Years	1
Genetic	Mitral valve prolapse	Pre‐existing	15 Years	1
Genetic	Hypertrophic cardiomyopathy	Pre‐existing	28 Years	1
Genetic	Arrhythmogenic right ventricular dysplasia	Pre‐existing	33 Years	1
CHD	Tetralogy of Fallot	Pre‐existing	2 Days	1
Genetic	Hypertrophic cardiomyopathy	Pre‐existing	3 Years	1
Genetic	Long QT syndrome	Pre‐existing	11 Years	1
CHD	Bicuspid aortic valve, patent ductus arteriosus	Pre‐existing	9 Weeks	1
Genetic	Left ventricular noncompaction cardiomyopathy		20 Years	
Acquired	Idiopathic cardiomyopathy	Antepartum	38 Years	1
Acquired	Pregnancy‐associated spontaneous coronary artery dissection (PSCAD)	Antepartum	39 Years	2
Acquired	PSCAD	Antepartum	34 Years	2
Genetic	Hypertrophic cardiomyopathy	Antepartum	33 Years	1
CHD	Patent foramen ovale	Antepartum	35 Years	3
Acquired	Peripartum cardiomyopathy	Antepartum	28 Years	1
Acquired	PSCAD	Early postpartum^a^	41 Years	3
Acquired	PSCAD	Early postpartum	37 Years	4
Acquired	PSCAD	Late postpartum^a^	36 Years	2
Acquired	PSCAD	Late postpartum	37 Years	2
Genetic	Hypertrophic cardiomyopathy	Late postpartum	28 Years	1
Acquired	PSCAD	Late postpartum	43 Years	2
Acquired	PSCAD	Late postpartum	39 Years	3
Acquired	PSCAD	Late postpartum	6 Years	3
Acquired	PSCAD	Late postpartum	36 Years	3
Genetic	Hypertrophic cardiomyopathy	Late postpartum	34 Years	2
Genetic	Long QT syndrome	Late postpartum	36 Years	1

Abbreviation: CHD, congenital heart disease.

^a^ Early postpartum within 42 days of birth; late postpartum up to 12 months following birth.

### 2.3 Data collection

Semi‐structured interviews were used because they are an established qualitative approach when exploring topics about which little is known, focus on the issues that are meaningful for the participant and allow for diverse perceptions to be expressed.^17^ An interview guide was developed, and data were collected via individual interviews conducted by phone. Interviews were conducted by a single interviewer (J. H.), took between 24 and 90 min and, with the women's permission, interviews were audio‐recorded or hand‐transcribed verbatim, including notable nonverbal responses such as crying or laughing. Personal details including names and addresses were not recorded with the study data. Information was reidentifiable only to the interviewer.

### 2.4 Analysis

Inductive reflexive thematic analysis was used as it is flexible and responsive when unexplored phenomena are described; allowed for nuanced theme development; facilitated the coding and organization of a large and complex data set; and it is able to highlight similarities and differences across the data set.^18^, ^19^ Informed by the six stages of analysis outlined by Braun and Clarke^18^, ^20^ (familiarization, code generation, theme development, reviewing and refining themes, defining themes and report writing), data coding and preliminary theme generation occurred concurrently with the interview fieldwork and were iterative and responsive to new data and developing patterns.

All study team members listened to the interviews and read the transcripts. J. H. led the analysis by immersing herself in the data and developing and refining codes and themes and selecting illustrative quotations. The approach to analysis was essentialist/realist (reporting on the experience, meanings and reality of participants), with semantic themes (reflecting the explicit content of the data).^20^

### 2.5 Study quality and research team

Each member of the team is a female healthcare professional (HCP) with diverse sexual and reproductive health and public health experiences. Our shared view is that PCC is ethically imperative and requisite for quality healthcare; thus, we approached this study believing that understanding and responding to patient experiences are important in ensuring positive outcomes for women, and we acknowledge that analysis in part reflects the authors' subjective interpretation.

Quality was determined using the guidelines provided by Braun and Clarke.^18^ In particular, the researchers engaged in ongoing discussion, reflection and development of the codes and themes, exploring individual and shared perspectives on the patterns within and across the women's stories.

## 3 RESULTS

The participants were diverse in age, diagnoses and timing of diagnosis; however, they all had rare, potentially life‐threatening conditions juxtaposed with the normality of pregnancy and postpartum that transcended differences. Analysis of the data generated five themes: (1) Dismissed: struggling to be heard, (2) Too little, too unclear: in search of information, (3) Winging it: research, education and guidelines, (4) Fragments: care co‐ordination and continuity and (5) Making do: fitting into services designed for others.

### 3.1 Dismissed: Struggling to be heard

The experience of feeling dismissed by HCPs was the most dominant individual theme in this analysis. Women with all diagnoses reported feeling dismissed in acute and chronic settings, in the community and in hospital, by medical staff, nurses, midwives, ambulance staff and allied health as well as by secretaries and practice managers of medical specialists. The participants suggested that this was due to individual HCP attitude, gender bias and due to a lack of knowledge about cardiac disease in women especially with pregnancy‐related conditions, in particular, ‘…because it's easy to dismiss a ‘healthy' young woman’ (P5).

The women felt unheard when pursuing an initial cardiac diagnosis and when they were experiencing new or ongoing symptoms for an existing diagnosis. Reports of subjective symptoms (e.g., shortness of breath and chest pain) and objective signs (e.g., electrocardiogram (ECG) changes and elevated troponins) were misattributed to other causes, often without adequate or any investigation. One woman presented with ‘a racing heart and cold sweat, aching arm, tight chest pain’, and had three positive troponins that the emergency department (ED) doctor concluded were false positives because she was a young female and ‘didn't fit a cardiac profile’ (P10).

Women's symptoms were most commonly attributed to as having anxiety, regardless of whether this was shown or expressed by the woman. Further, when women were told that they had anxiety, no referral or intervention was suggested to support the women.

*‘Do you feel anxious? You might be having an anxiety attack’ and I was saying to them, ‘No… It's not an anxiety attack’*. (P23)

The experience of feeling dismissed was iterative. One woman first presented to her general practitioner (GP) in late pregnancy and subsequently had multiple presentations over the following 8 months to both her GP and the ED with chest and jaw pain and ‘an odd heart rhythm’, where ‘they didn't even examine’ her before she was diagnosed with a myocardial infarction and multiple pregnancy‐associated spontaneous coronary artery dissection and had emergency bypass surgery (P6).

One woman reported seeking help for a decade for daily chest pain following her cardiac event. It was not until new research documented the phenomenon that she felt her pain was acknowledged as cardiac, and while she recognized the absence of research at the time, it was the ongoing lack of investigation or offer of support that concerned her.

*…the GPs, cardiologists, ER staff were all basically saying I was just being hypersensitive and that it was in my mind*. (P16)

There is an overlap of common cardiac, pregnancy and postpartum symptoms, with pregnancy and postpartum causes consistently foregrounded over possible cardiac issues apparently without any investigations. Women were told ‘You're tired; you've just had a baby’ (P8) without assessment. Even when women had a known cardiac condition and raised concerns, they at times felt unheard.

*…even with the breathlessness with me having a known cardiac issue*…*. I don't remember anyone ever actually just listening to my heart … they're just going to assume that its pregnancy related, not cardiac related*. (P4)

Common cardiac signs and symptoms were also attributed to other conditions such as scoliosis, dehydration, gastroenteritis or being overweight, again perceived by the women to be a lack of knowledge and a bias issue. A woman who experienced shortness of breath postpartum and later developed jaw pain, chest pressure and palpitations was told by her GP that her symptoms were because she was overweight and drank diet cola, and she was advised to lose weight. After 2 years of ongoing symptoms and multiple presentations to her GP, and once she had lost weight, her GP investigated, leading to a diagnosis of hypertrophic cardiomyopathy (P8).

A key consequence of women not being heard was delayed diagnoses and the associated preventable morbidity and emotional distress. The women were aware of the dangers inherent in not being heard and taken seriously and they felt ‘*angry*’ and ‘*disappointed*’ that they were ‘*dismissed*’ and ‘*fobbed off*’.

*To be honest, the way I was going, I think I could have just ended up dying in my bed and people would have still been saying ‘Oh, it's just the baby’*. (P25)

Concerns about prognosis and mortality risk were also under‐appreciated, and practical and psychological support was not provided. One woman and her partner asked their medical registrar to witness the signing of her Will and was told ‘Oh, you won't need this’ despite having just being told that the risk of dying during birth with her cardiac condition was *‘*50%–66%’ (P2).

The women felt that their pregnant status or having young babies was often not taken into account in care planning or treatment.

*They said ‘Well, you might not get an angiogram for a week or two’; I felt like I was very dismissed. I remember being in tears… snapping at one stage and saying, ‘Right, and so do these people have a little baby at home that they're breastfeeding?’. (P5)*

### 3.2 Too little, too unclear: In search of information

Information was essential for participants to understand their condition, inform decision‐making and to provide reassurance and confidence; ‘It just gives you a little bit of hope that somebody knows what the hell is going on’ (P3).

The women expressed frustration and concern at the lack of information and resources shared with them; they recognized that this was in part due to a lack of research but also felt that this was not the sole issue. Some felt that there was a perceived convention of withholding and gatekeeping information, ‘Some of the doctors are like, “I don't think she needs to see this”’ (P7). Other times, information was oversimplified or, alternatively, medical terminology was used and not explained.

*It was a bit frustrating because I didn't really get it, and everyone kept saying, ‘Dissection, dissection. Oh, this is the girl with the dissection’. I'm like, ‘What the fuck is a dissection? Can someone just say you've got a tear?’*. (P7)

Lack of information created a void that influenced the way in which the women understood the nature and severity of their condition and this in turn affected their ability to adjust and assume autonomy.

*I didn't think that what I had was serious… he just sort of gave me a brief description. (P8)*

Women wanted more information and sought it out through obtaining second opinions, online searches and support groups, and this led to both questions and answers.

*After* [reading] *these other SCAD sufferers, I've thought, ‘Oh, I wonder what an LAD is and where my tear was, and I wonder how much damage*?*’ I did know that I had an EF of 30, but I don't know what that means*. (P16)

### 3.3 ‘Winging it’: Research, education and guidelines

The women emphasized the need for more research and enhanced clinical training. They understood that their conditions may be rare in pregnancy and postpartum but also that this perception may be inaccurate.

*I keep getting told how rare this is, but… the more you learn about it everyone's starting to believe that it's not that rare, it's just really underdiagnosed*. (P9)

There was also concern regarding general knowledge, clinical assessment and reasoning skills, including being able to perform common assessments such as ECGs.

*I go into emergency and some of them have never even heard of what I've got… one in 500 people have this, that's really disappointing that some medical professionals have never heard of it, or they don't know how to treat it, or they treat it incorrectly*. (P8)

The women in our study perceived a lack of research‐informed clinical guidelines in cardiac, pregnancy and postpartum care and expressed frustration, disappointment and, at times, apprehension.

*The answers I was getting weren't really based on research or on best guidelines or, experience… no‐one could ever really give me real answers, and I felt a bit like that was just their gut feelings*. (P19)

The absence of guidelines meant that women and their healthcare providers spent additional time seeking information and guidance, often futilely.

*It was really hard that there wasn't any information out there, especially when it came to medication and breastfeeding…*[the GP and I] *kept having to ring up the hospital, a lactation information line, and then a pharmacist kept coming back and forwards*. (P10)

Women with pre‐existing disease reported a lack of clarity, consistency and communication about the optimal way to manage labour, birth and pain. Some women presented peer‐reviewed research on birth for women with their condition to their obstetricians and anaesthetists; however, their preferences were not followed, whether they sought a vaginal or a caesarean birth. This was felt to be partly due to a lack of clear research‐based guidelines, and that ‘…having access to their information or their guidelines for all obstetricians or cardiologists around Australia would be really helpful’ (P19).

### 3.4 Fragments: Care co‐ordination and continuity

Women with CDPP were managed by HCPs from a range of disciplines and specialities. Intra‐and interdiscipline co‐ordination was seen as inconsistent and was mostly experienced as lacking by the women, and this led to mixed messages, compromised communication, fragmented disrupted care and distress for women.

Some women proactively sought to enhance care coordination and communication, though this was usually unsuccessful. One woman consulted with her cardiologist, obstetrician, obstetric physician and the head of anaesthetics regarding birth, all of whom agreed that an epidural was safe; however, as she entered theatres, she was met by a different anaesthetist, who declined to administer an epidural due to ‘people with my heart condition having cardiac arrest’. As a result, she had to *‘… on the spot decide if I was going to go under a general anaesthetic to have my first child, or possibly risk cardiac arrest’* (P3). She understood the rationale, but was frustrated and distressed by the lack of communication and guidelines, and the futility of her efforts. Another woman tried to act as messenger and negotiator between specialists who she understood had not coordinated care.

*He* [the anaesthetist] *said that I should have an epidural and caesarean. I said that my cardiologist said that I couldn't have an epidural and he said ‘No, you can’. I said I couldn't and then he left*. (P20)

Another participant with pre‐existing cardiac disease chose to see a private obstetrician for the continuity of care that this would provide. She presented to hospital in early labour as per her obstetrician's advice; however, this plan was not communicated to the hospital and she was sent home, which she ‘thought that was pretty cavalier’ (P13). She gave birth at term on a weekend with the practice‐partner of her obstetrician who had not received any handover because her obstetrician said he did not expect her to give birth that weekend.

*I don't think it was a good enough reason not to hand the case over to whoever was covering*. (P13)

### 3.5 Making do: Fitting into services designed for others

Women described being ‘out of place’ regardless of what ward or service they were in. Those with a known heart condition were an anomaly in a maternity care setting, and pregnant and postpartum women were anomalies in cardiac, emergency and general wards, and the women perceived that this contributed to compromised care. Cardiac and rehabilitation services were designed for different populations. Specialist and multidisciplinary care were only available in major metropolitan centres, reducing access and increasing the cost, stress and time required for women to attend. There was little or no service design modification to accommodate pregnant women and women with babies and small children. Specialist obstetric physicians were only available in a few hospitals.

The women recognized that health professionals do not specialize in multiple areas, but at times felt concerned about the care received. In maternity wards, ‘the staff…don't have a huge knowledge on the impact of cardiac illness on pregnancy and afterwards’ (P2). In cardiac wards, staff were not used to caring for pregnant and postpartum women: ‘that was a huge concern. I don't think that they looked at me as a pregnant woman. I think they looked at me as a cardiac patient’ (P16). The lack of knowledge and clinical guidelines in speciality areas was amplified when they were in other wards.

Mixed gender wards were particularly difficult for new mothers, such as the following woman admitted after an acute cardiac event postpartum.

*I was in a shared room with older men with only a curtain between us, I'm having to sit there breast pumping, I've got my newborn in there … it was a pretty horrendous experience, the whole thing*. (P12)

Ward management practices appeared to inconsistently reflect baby‐friendly hospital practices. Some women were advised that their baby could board in the maternity ward, or that their partner could bring the baby in to her for feeds, presumably not hourly. Not only was the option of having their baby stay with them not always available, but women felt scolded for asking. The following woman was in a hospital that had a maternity unit.

*There had been a whole lot of kafuffle where they'd said, ‘It's fine, the baby can stay’, because he's fully breastfed, and then the [manager] was, like, ‘No. He cannot stay. Don't be ridiculous’*. (P23)

Cardiac rehabilitation was an area that was consistently reported as inadequate or simply unclear for women with CDPP. Women with the same condition were variously advised to do rehab, avoid it or that it did not matter either way. Most women felt that rehab as it was offered was not relevant for them. All women who attended rehab noted that it was not designed to accommodate mothers with babies and that the physiotherapist or nurse facilitating the sessions did not always have knowledge of the women's conditions. Some women attended rehab to regain confidence, where others wanted specific guidance that was not provided.

*I felt I just wasted my time. There was nothing about exercise restrictions or what I should be doing. (P10)*

For some women, the experience lessened their confidence as they were excluded due to being symptomatic, even though having ongoing symptoms was their ‘norm’.

*I did the cardiac rehab, which I was kicked out of, because I was experiencing pain and they were too scared…I don't think they understood; they had no idea what SCAD was. I had told them that I experienced the pain, whether I sat down, lay down, did exercise, what not. But they weren't comfortable with me maybe dropping dead in their care, I presume. (P16)*

The limited written or digital material available was perceived as irrelevant by most of the women, and ‘…the only real support services related to the heart are for people with [atherosclerosis]’ (P13). The lack of resources was especially felt in the absence of a pregnancy and mothering framing, and availability of age‐ and disease‐specific support groups.

*Where does a 25‐year‐old pregnant woman go who's been diagnosed with a heart condition? There's no real support network for that. (P22)*

The above analysis captures the most compelling and consistent themes generated from the data; however, it is important to note that this is not the totality of experiences. When women felt heard, it made a profound difference: ‘You two have been like the first doctors I've really trusted because you've actually listened’ (P1). Some women had supportive, informative and respectful interactions where they did not ‘… feel like I'm going to be belittled by asking’ (P7). Finally, an example of coordinated care was a GP sharing woman's hospital discharge summary with the other GPs in the practice so that everyone was aware of her history and how to manage her care if her treating GP was absent.

## 4 DISCUSSION

This study explored the healthcare experiences of women with CDPP and found that their healthcare expectations and needs were not being fully met. The majority of women in our study described a spectrum of largely negative healthcare experiences across multiple presenting cardiac conditions.

### 4.1 Feeling dismissed

The patient experience of being ‘dismissed’ has been documented in areas of health relevant to women with CDPP including reproductive health, cardiac disease and rare or medically unclear diagnoses.^21^, ^22^, ^23^ Women in our study felt dismissed when presenting at their GP and the ED as well as during pregnancy and during labour, increasing the risk of missed or incorrect diagnoses, morbidity and potentially death. All women experienced delays in diagnosis, and or responding to deterioration in pre‐existing cardiac disease, similar to previous findings of it taking 3–190 days for women with peripartum cardiomyopathy (PPCM) to be diagnosed.^24^

In our study, feeling dismissed affected the women's perceptions of HCPs and, in some cases, reduced trust and decreased the likelihood of compliance with treatment or follow‐up. Our study is consistent with earlier work that found that nearly 40% of women with PPCM experienced symptom dismissal by HCPs and 25% were initially given inaccurate diagnoses, including ‘new mum anxiety’.^25^ Analysis of posts on a PPCM online support group similarly reported that women were ‘brushed off, dismissed and ignored’, and incorrectly diagnosed, including with anxiety.^26^

### 4.2 Person‐centred care

The experience of feeling or being dismissed is counter to the tenets of patient care and PCC. In patient‐centred care, the patient participates as a respected and autonomous individual, and care is based on individual patient's physical and emotional needs.^27^ Almost all of the women interviewed described a lack of patient‐centred care. PCC is broader and includes the needs and expectations of families and communities and their role in shaping health policy and services and incorporates individuals' personal social determinants of health.^28^

The Institute of Medicine (IOM) published its six dimensions of patient‐centredness as essential to providing quality healthcare more than 2 decades ago.^10^ These dimensions are that care needs to (1) be respectful to the individual's values, preferences and needs; (2) be coordinated and integrated; (3) provide information, communication and education; (4) ensure physical comfort; (5) provide emotional support; and (6) involve family and friends.^10^ Reflecting on the healthcare experiences of the women in our study, it is clear that the goals of PCC are yet to be realized, with shortfalls apparent in each of the dimensions.

Women in our study variously felt that they were viewed as ‘all baby’ or ‘all heart’. but never as a whole person or mother. They described that their needs both within and beyond the hospital setting were not recognized or responded to. They felt that they were seen as a diagnosis and not a person, and did not feel included in decision‐making. Pregnant women, women during labour and birth, women with complications during pregnancy and women experiencing acute cardiac events as new mothers all felt vulnerable, lacked autonomy and struggled to receive the information that they required to engage in their own healthcare decisions. Their needs as pregnant women or as mothers were not consistently included in the care provided.

PCC and self‐advocacy require effective bidirectional communication, which was lacking in the majority of women's experiences. Instances where women attempted to self‐advocate and navigate the health system were mostly unsuccessful; however, communication and ensuring safe and effective PCC should not be the burden of those with the least power, the patients.^29^ To successfully self‐advocate in health, patients require three attributes: support systems, effective communication with disparate HCPs and the ability to critique and use health information.^30^, ^31^ In addition to the heightened vulnerability of having a potentially life‐threatening cardiac illness in pregnancy and postpartum, the women in our study had rare conditions, with little information or support available, making it difficult to self‐advocate and manage their health experience. Not acknowledging women's knowledge of their bodies, symptoms and needs exists in a sociopolitical context of devaluing women's knowledge and lived experiences, including of illness.^32^, ^33^

Continuity of care and care coordination was a priority for the women, but was mostly experienced as inconsistent or absent. Continuity of care relates to quality care over time, reflecting the extent to which a series of discrete healthcare events is experienced as coherent and interconnected, and compatible with the patients' health needs and preferences. Data show that continuity of care and care coordination is highly valued and is central to PCC; it facilitates trust through ongoing relationships with HCPs and reduces ED visits, hospitalizations and overall health expenditure.^34^, ^35^

Women with CDPP often have long‐term complex care that requires the involvement and coordination of care from multiple HCPs across different specialities and disciplines. The results of this study reflect those of Hinton and they highlight gaps in coordination and continuity of care, leading to fragmented and inadequate care for women who presented with CDPP.^36^ At the time of writing, there are only a few cardiac obstetric clinics in Australia and not all hospitals offer both maternity and cardiac services or have obstetric physicians. Further, the women in this study experienced little involvement of anaesthetics services in planning care. This lack of co‐located obstetric and cardiac services may jeopardize communication and co‐ordination between teams.^37^ Even when women in our study were assertive and proactive, their attempts to coordinate their own care and act as liaison between HCPs of different disciplines failed.

### 4.3 Healthcare professionals

Working within a PCC framework is dynamic, necessarily lacks definitive protocols and potentially requires a shift in practice for HCPs and patients and a reimagining of societal perceptions and expectations of HCPs. While PCC is seen as especially important for vulnerable groups, it may be less accessible during serious heath events involving rare and uncommon conditions, which may result in reduced provision of information and shared decision‐making, ultimately leading to the individual not receiving PCC, as seen in our study. Clinicians working across specialty areas may feel less confident or competent in some areas and need education, support and guidelines to facilitate best practice, avoid burnout and tailor management that acknowledges patient experience, especially for long‐standing and complex conditions.^38^, ^39^

### 4.4 Healthcare system

It was not our intent in this study to seek fault in HCPs, and for balance, some women described feeling heard, believed and supported and this made a profound difference to their experience of clinical care and the level of trust and safety they felt. However, we note that many of the examples that women provided of excellent PCC often involved HCPs going ‘above and beyond’: an obstetrician checking in whilst on leave, the obstetric physician's negotiated risk assessments to respond to a woman's request, the midwife staying back hours after her shift ended to provide continuity during labour, the cardiac nurse drawing diagrams at 3 am to ensure that the woman understood her diagnosis, the cardiologist providing extended access and the GP spending additional time trying to find elusive answers when trying to support the woman's desire to continue breastfeeding. These examples highlight good practice, but the most critical feature is that these examples are of HCPs providing care that is not integrated into regular care, and not structurally or financially supported within the healthcare system. In presenting the six dimensions of PCC, the IOM identified the need to build organizations and systems that support change.^10^ PCC encompasses more than clinical interactions and we cannot rely on the professionalism, empathy and excellence of individuals to provide PCC *despite* the system within which they work. We need a healthcare system that carries the burden of implementing PCC and enables HCPs to deliver excellent care. The findings of this study add to recent work exploring patient‐ and HCP‐identified outcome measures for CDPP, and strategies to implement PCC.^40^, ^41^, ^42^

## 5 LIMITATIONS AND STRENGTHS

This study may be subject to both positive and negative recall bias. The generalizability of our findings is limited to English‐speaking patients, with no representation of Australian First Nations women or minority ethnicities. More studies are needed to understand the specific needs of women with CDPP, including the needs of diverse populations and needs over time.

A strength of this study is that this is the first study to explore women's healthcare experiences across a spectrum of CDPP. This knowledge contributes valuable information to a small body of knowledge on women's experiences and values relating to CDPP. The interviews allowed women to be authentic and share what was of most importance to them. There was an intensity and density of themes, especially regarding being dismissed, lack of clinician and patient knowledge and the need for PCC.

## 6 CONCLUSION

Studies on women's healthcare experiences are essential to build patient agency, healthcare knowledge and inform care. This study identified a lack of PCC for women with CDPP. Of concern is that this equally applies across pre‐existing and de novo diagnoses, reflecting a lack of responsiveness of the healthcare system to providing fit‐for‐purpose healthcare for women with complex chronic disease who are pregnant or new mothers. This study identified a number of areas in which women wanted system improvement, including treating women with respect by listening to them, multidisciplinary care planning and co‐ordination, increased clinician knowledge and competence and investment in clinical guidelines, research and patient support. There is an opportunity for cardiac and maternity care providers to listen to women about their healthcare needs and build upon their experiences to enhance care for women with CDPP.

## CONFLICTS OF INTEREST

The authors declare no conflicts of interest.

## Data Availability

Research data are not shared. The data are not available due to privacy or ethical restrictions.
